# Pathology in Dental Practice

**Published:** 1901-10-15

**Authors:** George W. Cook

**Affiliations:** Chicago, Ills.


### PATHOLOGY IN DENTAL PRACTICE.

BY GEORGE W. COOK, D.D.S., CHICAGO, ILLS.

Abstract of paper read before the National Dental Association, Milwaukee, August 6-9, 1901.

We have a number of ill-defined symptoms supposed to arise from the non-elimination of cellular excretory products or the non-oxidation of certain nitrogenous matter. This is called autointoxication. Primarily, this conception was based upon the fact that all living plant and animal life excretes certain substances which are poisonous to the cell from which they came. The question naturally arises, where does this animal excretion originate; it is not taken in with food or drink; for if one took nothing but pure food, pure water, and breathed only pure air, the excretions of the organs are not found in the normal tissues of the body. In the presence of warmth and moisture, proteid matter is broken up by bacteria into its ultimate simple molecules, nitrates, nitrites, ammonia water, and the various inorganic elements, viz., S. Fe., Na. K. Mg. Ca., etc.

During this process of splitting up the protein molecule by bacteria, there are a number of intermediate products formed which are basic nitrogenous crystalline substances, and are intensely poisonous. These are called ptomains, the formation of which always points to action of bacteria on proteid substance.

In the chemical process of splitting up the proteid molecule of the cell, there is formed a comparatively simple molecule constituent, known as urea. The proteid molecule, when split up in this way, necessarily gives rise to the formation of a number of intermediate products which are looked upon as steps in the oxidation process; they are antecedents of the nitrogenous end product urea. Among these might be mentioned certain of the amid compounds: Adenin; hypoxanthin; xanthin; guanin; uric acid, etc. These compounds which were supposed to originate in the tissues either along with or a precursor of urea and uric acid, are termed leucomains.

As for their being poisonous to the body, we can no more admit than that caffeine is poisonous to the coffee tree, or that nicotine is poisonous to the tobacco plant, for in both cases these alkaloids exist, not as pure xanthin or caffeine, but in the proteid compound which renders them non-toxic to the plant or individual in which they are found. But the idea is still prevalent that during the process of oxidation—by this term we mean the transformation of the complex proteid molecule as it exists in the living cell or the dead food material, to a more simple compound eventually ending in urea water and excreted from the body as such—certain of the supposed intermediate products are no further oxidized and remain in the tissue and are capable of producing toxic symptoms. That proteid matter is excreted from the animal body in the form of urea is true, but we have little or no evidence that certain basic substances in dead proteid matter are intermediate oxidation products. It is well known that if urea be retained in the body, toxic symptoms will be produced (uremia), in such a case, the term autointoxication might be implied.

Under normal conditions the animal body as a whole is able to dispose of its own excretion through the bowels, kidneys, and skin, likewise every cell is able to contribute its normal quota of excretory matter to general avenues of excretion, viz., kidneys, bowels, etc., cease to do their work and toxic symptoms will soon develop. Going still furthcr back the individual cells may cease to excrete their dead matter and also cause retention followed by toxic symptoms. But the primary cause which so changes the chemical constituent of the individual cells so that they no longer carry on their work in a normal way, or so change the kidneys that they no longer excrete the normal amount of urea from the body, or produce poisonous decomposition products in the bowels, must necessarily come from without; some agency acting as a disease stimulus. This view must then necessarily do away with the term autointoxication, or the autogenous disease as a primary pathological entity and reduce such conditions to the plane of mere secondary conditions following the external disease stimulus.

We have many reasons for believing that many of the so-called autointoxications or autogenous diseases are due to a chemical change in the proteid molecule of the cell which is caused by a chemical change in the cell environment.

This change must necessarily bring about a change in metabolism, thus liberating the poisonous products of the cell. But where must we look for the primary cause? In nature all organic matter is reduced to its primary elements by the agency of bacteria; this is the enzymotic or fermentative action of bacteria. Also on living organic bodies certain bacteria find a food media and act in an enzymotic or fermentative way, giving rise to certain chemical changes in the cell.

So far as we know, we can trace all so-called autointoxication to a primary sub-infection from the bowel or respiratory tract. Bacteria may act any place along the alimentary tract from the mouth to the lower bowel. If the condition of the mouth is not hygienic or the food is not of the proper kind, the stomach and bowels can not dispose of it at the right time, thus giving lodgment to various fermentative bacteria; they or their product are readily absorbed into the system, either of which may give rise to poisonous symptoms or may so change the chemistry of the blood lymph of the cells themselves, and also change non-pathogenic to a pathogenic bacteria, which are generally looking for an entrance into the body either through the respiratory or gastrointestinal epithelium, there finding a favorable media in which to grow and carry out their further changes which are incompatible with the life of the host. This second condition is really a sub-infection and really explains the ways in which all infectious disease may originate, whether of a local or general character.

When we consider that a disease process is the result of a series of factors, which are of unequal value, we are then forced to say that the essence of the disease is not in the cell (Virchow), neither can we say it is time or place (Pettinkofer) or a bacterium is the true and sufficient cause of disease (Koch). But all of these are factors of unequal value.

We can no longer say pyorrhoea alveolaris is constitutional any more than an alveolar abscess is constitutional, both of which are to be dealt with as local microbiological affections, the same as rheumatism, catarrh, influenza, pneumonia and diphtheria. The cure of this disease, as of all other infectious diseases, must be to remove the predisposition of the individual under which come the phenomena of inherited and acquired immunity and predisposition to this disease.

The pathogenic properties of micro-organisms in the saliva depend upon the nutritive value; therefore, it is purely the chemical properties of saliva that manifest themselves in the changed condition of micro-organisms, both morphlogically and pathologically. In this connection I will relate some personally tabulated observations of persons whose gastrointestinal disturbance was the predisposing cause of local infection.

Vincent described what he called the bacillus fusiformis (which is quite common in the human mouth), and called attention to certain pathological processes on the oral mucous membrane. I have had an opportunity of studying the vital phenomena of this micro-organism. It seems to live in the oral cavity as a saprophyte until intestinal disarrangement appears, then, if this trouble is but slight, this bacillus will set up only a passive inflammatory process. Should the intestinal trouble increase, the local ulceration also increases. It is only with a bacteriological examination that it can be determined from some of the syphilitic lesions in the mouth. I am not prepared to say, as yet, whether or not this germ is always present in the oral cavity; but if it is, there are times when there are but few.

The point I wish to bring out is that this germ may be considered as one of the oral cavity, for the reason as soon as any gastrointestinal trouble is set up mucous patches may appear on the oral mucous membrane or tonsil, possibly both, in which it is the exciting cause. I believe, as Lesner does, that in mercurial stomatitis it becomes a factor of considerable importance; by local treatment the local affections disappear, but in a few days it will return unless the predisposing cause has been removed. How true is this in pyorrhea alveolaris? There are at least six other affections of the oral mucous membrane, which are caused by the action of micro-organisms, but only when there is a predisposing cause in the body fluids. Marmorek, Kolle, Widel, Bezancon, all have observed the difference between streptococcus grown in the mouth of healthy persons and those grown under other circumstances. Those grown in the mouth are short chains and will not set up a disease process as those grown in bouillon.

In six cases of rheumatic persons I studied bacteria of the tonsils and found that three of them had a diplococcus described by F. Myer. All of these cases have pyorrhea alveolaris, and showed the characteristic bacteriological finding. This diplococcus found in the tonsil in some cases could be called a streptococcus. The length of the chains seems to depend upon the conditions of the gastrointestinal tract. When the bowels become constipated, it manifests itself in the length of the chains taken on a streptococcus form. When salian cathartics are given internally, or if the individual is put on sodium salicylate, the dipplococcus forms again appear, and finally disappear from the tonsillar mucous. Clinicians have observed such conditions in cases of the injection of antitoxin.

In conclusion I wish to emphasize the point that there is a marked line between a predisposing and an exciting cause of disease.

### DISCUSSION.

Dr. W. H. Whitslar, of Cleveland: Were it not for the statement of the essayist that poisons within the body are not due to external stimuli, I should feel that the request of a few minutes ago, that I open the discussion of this paper, had produced within me auto-intoxication. Physiological knowledge is necessary to determine where health leaves off and disease begins. There is a natural resilience of the normal functions, but an excessive diviation due to one or more of the many causes will produce continued functional aberration, or disease. The essayist well says that disease is not dependent on disturbed cell function alone, but cell tissue and organic function may all be influenced by the excessive energy of the activity of various micro-organisms. The chemico-physiological functions are peculiarly susceptible to the influence of micro-organic intoxication.. The saliva may be secreted under such influences as will give it a healthy or unhealty function. It may assist or retard digestion. It may render the mouth sterile or the reverse; and in exactly the same way the liver, kidneys and other organs may singly or in combination assist to disarrange the physiological functions, producing what the essayist has termed subinfection. The nervous system, which controls all the vital organs, is the vital point of attack. By irritation of the nerve function in a single organ the entire nervous system may be greatly influenced, and when there is a combined attack from many different, or contiguous organs, the nerve disease becomes more serious, and if the primary stimulation be not sufficient to throw off the disease, general prostration ensues. From this it will readily be seen that this is a most important subject for even the specialist who only treats a limited area of the whole body, and yet that portion may be influential in producing great systemic consequences.—*Ohio Journal.*

